# Industrial Dentistry*Read before the Cincinnati Dental Society, January, 1920.

**Published:** 1921-02

**Authors:** J. P. Becker

**Affiliations:** Cincinnati, Ohio, Consulting Industrial Dentist with the R. K. LeBlond Machine Tool Company


### INDUSTRIAL DENTISTRY*

BY J. P. BECKER, D.D.S., CINCINNATI, OHIO
Consulting Industrial Dentist with the R. K. LeBlond Machine Tool Company

In looking at the subject of Industrial Dentistry, the first question which presents itself is, "Where does industrial dentistry belong in the scheme of production?" The importance of this question lies in the fact that until we have oriented it, until we have assigned it its proper place in the productive process, we are in no position properly to evaluate it either socially or economically. This evaluation is necessary if we are to determine either its present worthfulness or its future development.

Is industrial dentistry merely a phase of welfare work, is it something done for the worker by the employer? If this be all that it is, then its main value will be social and therapeutic ;and in attempting to estimate this value we must ask what is its reaction upon the workingman, how does he look at it as a piece of welfare work and what is its therapeutic value?

Assuming that it is done by skilled and scientifically-trained operators, who have all needed facilities for doing the work, all adjuncts to proper diagnosis and ample time in which to do the work, we may accept its therapeutic value as unquestioned.

But this is only part of its total social value, the other and possibly more important part is the worker's reaction to it as part of what we are pleased to call welfare work. It is rather important for us in these days of industrial unrest to inquire rather closely as to just how the workingman looks at all this thing which is denominated welfare work, and in which industrial dentistry may or may not be included.

* Read before the Cincinnati Dental Society, January, 1920.

Pure welfare work, and by pure welfare work I mean that work done for the employee by the employer and at no expense, either immediate or remote, to the employee, was undoubtedly motived in the great majority of cases by a sense of social obligation upon the part of the employer to the employee, and a desire to do something for the employee in addition to merely paying him an agreed wage. In all of its varied manifestations it has been an attempt to make the life and labor of the employee more safe and comfortable, more pleasant and enjoyable, more happy and satisfactory.

Looked at from the viewpoint of the one instituting and supporting this kind of work, it is altogether admirable, and he naturally feels that he has a right to expect that it will be received by the beneficiary of it and accepted by him in the same spirit in which it is given; and he is not only pained, but also sometimes shocked when he realizes that the employee frequently not only fails in gratitude but is actively antagonistic to the whole idea of welfare work.

That the employee does have this feeling of antagonism has been demonstrated repeatedly by both word and deed. In many cases he resents all kinds of welfare work and in others he resents simply one kind. We will consider this differentiation between kinds of welfore work later on in this paper, and confine ourselves now to the reason why the employee may not approve of purely gratuitous work done for him by the employer, and we consider his attitude because the social value of the work largely depends upon this very factor. No work can be socially valuable which does not result in the betterment of relations between the one doing it and the one for whom it is done.

If the workingman resents it because he feels that it is unduly paternalistic, or because in the final economic analysis he is really paying for it himself, or because he dislikes having anything done for him without his paying for it, or for any other reason whatsoever, then it is seriously to be questioned as to whether such work is socially valuable and whether it should be continued or discontinued. That the workingman does, in many cases, at least look askance at all welfare work, even if he be not actively opposed to it, is capable of demonstration by the very simple process of securing the opinion of a sufficiently large number of them to give you a basis for judgment.

If an appreciable proportion of workingmen resent the idea of welfare work, then industrial dentistry, as a part of this kind of work, has at least a questionable value as far as its contribution to social well-being is concerned.

On the other hand, we may ask, is industrial dentistry an effort upon the part of the employer to reduce the cost of production by increasing the physical efficiency of labor?

If this is what it is, then it is in no sense welfare work and our criterion for evaluating it must be purely economic rather than social. But whether it be welfare work or an economic expedient for reducing the cost of production, there is one question which must be asked and answered, for upon our answer to it depends the justification of the workers' attitude, and both its social and economic value as part of the productive process.

This question is, who pays for it, who bears the expense of industrial dentistry as it is generally carried out?

There are at least four possible answers to this question and each answer carries with it different results.

In the first place, industrial dentistry may be paid for by the employer and may be a gratuity to the employee from capital's share of the net product of industry.

If this be the case, then the employee may have grounds for a just resentment against industrial dentistry as being charity, something given to him for which he makes no return, and which places him in the position of one who is the recipient of alms, and he may rightfully resent being placed in this unenviable position, and thus the whole social result of the practice may be harmful.

This social harmfulness also would tend to affect adversely any possible economic gain which might result from this particular method of consumption of his wealth upon the part of the employer. A dissatisfied workman is never at the top-notch of his efficiency, and any decrease in efficiency means increase in cost of production, and wherever such increase is unnecessary it is economically unjustifiable. Not only is this true, but it may be possible that the employer may feel that this gratuity upon his part must, at least partly be made up by retrenchment in some other particular or particulars, and this retrenchment may be felt by the workingman as disadvantageous to him and thus, even as a pure gratuity, industrial dentistry may be economically harmful.

In the second place, industrial dentistry may be paid for by the worker, provided the wage fund is reduced by an amount equal to the cost of the treatment.

If this be the case then there is no question as to who should do the deciding as to whether we should or should not have such a thing as industrial dentistry, and the question of its value will be determined by the one paying for it. In this case the workingman must make the decision and must determine whether or not there shall be such a thing as industrial dentistry.

In the third place, it may be paid for by the consumer of the finished commodity, provided the amount thus spent on it is added to the selling price of the article manufactured. Here a new factor enters, *i. e.*, the consumer, and in this case it seems but logical that he should have something to say about whether or not there should be such a thing as industrial dentistry. Surely, if he pays for it, he ought to be given some say-so about whether or not he wants it. But the realization of justice to the consumer is an iridescent dream, and the fact that he pays for a thing has only an academic interest to everyone but himself and, besides, he has grown so used to paying that he might even resent a reduction in price as being discriminatory against his ancient and vested right of being forced to pay without having any determinative voice in fixing the amount which he has to pay, or to whom he must pay it.

In the fourth place, it may be paid for by both capital and labor, in case a profit-sharing scheme is in operation and the expense is listed as part of the cost of production. Here three parties should rightfully have some part in determining whether there is or is not to be industrial dentistry, viz., the employer, the employee and the consumer, and the social and economic results will depend upon the answer which they give, and its effect upon any or all of them.

The question as to who pays for industrial dentistry is important because upon the answer to it depends the answer to the other question, as to who should determine whether there shall be such a thing as industrial dentistry. If industry is conducted upon a purely capitalistic basis, and labor has no representation in its control, then, whether this method of conducting production be right or not, the capitalist would be the man to determine whether there should or should not be industrial dentistry. If both capital and labor are represented in the control of industry, then both should have a share in determing the advisability of having or not having industrial dentistry.

If capital, labor and the public have a share in the control of industry, then all three should share in the determination.

Another question which is worth while thinking about is, who should determine the manner of the administration of industrial dentistry?

Here again the answer depends upon control of industry. It is remarkable how most questions affecting industry do depend upon this question of control, and how slow we have been to realize that this is the case and to take it into consideration in our thinking about things.

If the capitalist exercises exclusive control over industry he would normally determine the administration of everything connected with industry. His determination would of course be affected, if he were wise, by the advice of experts and the psychologic reaction of labor to the plan.

If labor shares in control, then labor would share in determining the manner of the administration of the scheme.

The whole thing comes back to the question of control and this matter of control is basic and fundamentally important, for if it be overlooked, friction and misunderstanding are bound to result, and to the extent that either exists there will be an interference with the smooth and easy working of any plan for either social or economic improvement.

You will find too, I believe, that the question of control affects the attitude of labor to all questions which have to do with welfare work or with anything else which affects him in his industrial relationships.

What is labor's attitude toward the whole question of industrial dentistry?

If industrial dentistry be merely a phase of welfare work and not charged against the cost of production, I believe that many workingmen will resent it because of its charitable implications, and because they feel that they should have a voice in determining whether or not it should be put in practice because they are the ones most directly affected.

If it is an effort to reduce the cost of production by increasing the physical efficiency of labor, then I believe that many workingmen will resent it because it savors of the idea that labor is something separate and apart from the man, and that its efficiency or non-efficiency is to be determined without consultation with the man who supplies the labor. This may be a shortsighted view upon the part of labor, but the fact remains that the laboring man fiercely resents anything which tends to depersonalize labor.

If it is a phase of welfare work and is charged against the cost of production, then the laboring man has the right to question its installation, because it must be paid for out of the totality of returns from which all costs must be paid and from which all profits and wages and rents and salaries must come. Any deduction from this totality he looks upon as being in part a deduction from what might otherwise have gone to increase the wage fund.

If there is non-profit-sharing scheme in force in the industry, then the laboring man looks upon all welfare work as probably being paid for largely by himself and feels that if it were not in existence, there would be more money for wages and it is in wages that he is most vitally and constantly interested. Therefore he resents welfare work.

If there is a profit-sharing arrangement of any kind, then the laboring man feels that he is entitled to have a voice in the determination of policies which involve the expenditure of money earned by the industry, for every dollar spent which does not mean an increase in production results in diminishing the profits and consequently in decreasing labor's share.

All these questions have a direct bearing on the subject as to whether industrial dentistry is either socially or economically justifiable as it is conducted today, and our judgment on the broader subject will depend upon the answer which we give to them, but there is a still more fundamental question which it behooves us to consider rather carefully, and this is a question which strikes directly at the root of the whole matter and raises the issue as to whether it is right that private industry should be burdened with conserving the health of its employees by giving them dental attention, or is it a legitimate function of the state to do this, and this is but a phase of the yet larger question as to whether all health measures should not rightfully be under the immediate direction of the state and supported and paid for by the state.

If private industry looks after the health of its employees and spends thereon large sums of money, and this result can not be accomplished without spending large sums, then the consumer ultimately pays for it, provided that it does not so reduce the cost of production as to render the finished commodity cheaper in the open market.

As to whether or not it does reduce the cost of production to such an extent as to pay for itself is a question to be decided in each individual case by a careful examination of costs.

If it does not so reduce the cost of production, and the additional cost involved is charged against the selling price of the commodity, then the consumer is taxed without his assent for a benefit conferred upon but a part of the people, and in which he does not share except as an unwilling and involuntary disbursing agent. It is open to question as to whether this is right and just.

The consumer rightfully resents the idea that he is being exploited in order that additional advantages may accrue to the employer and he is growing increasingly restive under the pressure of rising prices. Is there any difference in principle between the exploitation of the consumer for the benefit of the employer and his exploitation for the benefit of the employee? And, unless industrial dentistry result in an economic gain, is it not a charge against the consumer in favor of the employee, provided that the total cost of it is not borne solely by the employer or shared in its entirety between the employer and the employee?

After all, is not the only equitable and just solution of the whole problem for the state to take over all matters concerning the health of its citizenship? The state is already exercising numerous health functions and the tendency is toward their constant extension and expansion. We have boards of health which are constantly enlarging the scope of their work; we have great public hospitals where the poorest in the land can have treatment and care which is possible only to them and to the very rich; we have public food inspectors; we exercise public control over contagious diseases; we insure an adequate supply of pure water, sometimes at great expense; we have public health stations and public district physicians and free clinics of various and sundry sorts.

The increasing functioning of the state along these lines has come about as a result of an enlightened public demand, and the public is becoming increasingly ready and willing to bear the necessary burden of taxation incident to their support, provided that it be convinced that the work is being carried on both efficiently and economically. These are comparatively new functions and have but recently been taken over by the state from the hands of private enterprise. Their development is more or less paralleled by that of the public school system. There was a time when the state was willing to entrust the mental health and development of its future citizens to the hands of private individuals who operated schools for the benefit of those able to pay for the education of their children. The system of private schools for elementary education has been almost completely discarded and no one would now be so foolish as to suggest that we revert to it. We are satisfied that education should be a function of the state, and if the insurance of a sane, normal, mental development logically belongs to the state, and if the state has demonstrated its fitness to assume this burden for all of its citizens, then why not assume the simpler and equally important function of, so far as it is scientifically possible, assuring the health of all of its citizens?

Dr. Charles Mayo says most of the diseases from which we suffer are but symptoms arising from primary foci of infection found in the oral cavity, and nine out of ten will die from a streptococcus infection. The late Dr. Osler has said that oral infection has done more harm to humanity than strong drink. These conditions arise from a lack of knowledge of oral and general hygiene, which we believe can be to a great extent corrected by properly-imparted instruction. The free dental clinic secured the services of an expert teacher of oral hygiene, Dr. Salmons, whose work during the past winter has been the means of arousing an increased interest among those who formerly gave little thought to the dangers arising from pathological conditions of the oral cavity.

In my opinion public health is of coequal importance with public education and most intimately connected with it, and if the one be entrusted to the state, so should the other.

We seek to restrain by legal enactment the practice of dentistry by those not properly educated and we provide public examining boards for those who desire to enter this profession. This in itself is an admission that the state is concerned with the health of its citizens and that it admits its responsibility and assumes its obligation.

Why not take the last step in this logical progression and have dental education paid for by taxation, and given by competent, scientifically-trained dentists who shall be responsible to the state for the proper performance of their duties?

When this is done, all dental welfare work will become a thing of the past and industrial dentistry will be but a phase of public dentistry, and the burden will be borne by all the people, for we shall recognize that all the people have the right to expect and demand from a democracy that it shall make itself responsible, so far as is humanly possible, for the physical as well as the mental health and well-being of all of its citizens.

